# Association of sexually-transmitted infection and African–American race with *Streptococcus agalactiae* colonization in pregnancy

**DOI:** 10.1186/s13756-020-00827-1

**Published:** 2020-11-04

**Authors:** Gerald A. Capraro, Sajel Lala, Khaldia Khaled, Elizabeth Gosciniak, Brianna Saadat, Sarah M. Alvarez, Seema Kumar, Tara Calhoun, Edward Landry, Gloria Caldito, Joseph A. Bocchini, John A. Vanchiere

**Affiliations:** 1 Department of Pediatrics, Louisiana State University Health Sciences Center – Shreveport, 1501 Kings Highway, #5-303, Shreveport, LA 71130 USA; 2 Department of Pathology and Translational Pathobiology, Louisiana State University Health Sciences Center – Shreveport, Shreveport, LA USA; 3 School of Medicine, Louisiana State University Health Sciences Center – Shreveport, Shreveport, LA USA; 4 Department of Neurology, Louisiana State University Health Sciences Center – Shreveport, Shreveport, LA USA

**Keywords:** Group B *Streptococcus*, Pregnancy, Inducible clindamycin resistance, Azithromycin, Sexually transmitted infections

## Abstract

**Background:**

Group B *Streptococcus* (GBS) remains a significant cause of neonatal infection, but the maternal risk factors for GBS colonization remain poorly defined. We hypothesized that there may be an association between antibiotic exposure during pregnancy and GBS colonization and/or the presence of inducible clindamycin resistance (iCLI-R) in GBS isolates from GBS-colonized pregnant women.

**Methods:**

A retrospective cohort study was performed at Louisiana State University Health Sciences Center – Shreveport including demographic and clinical data from 1513 pregnant women who were screened for GBS between July 1, 2009 and December 31, 2010.

**Results:**

Among 526 (34.8%) women who screened positive for GBS, 124 (23.6%) carried GBS strains with iCLI-R (GBS-iCLI-R). While antibiotic exposure, race, sexually-transmitted infection (STI) in pregnancy, GBS colonization in prior pregnancy and BMI were identified as risk factors for GBS colonization in univariate analyses, the only independent risk factors for GBS colonization were African–American race (AOR = 2.142; 95% CI = 2.092–3.861) and STI during pregnancy (AOR = 1.309; 95% CI = 1.035–1.653). Independent risk factors for GBS-iCLI-R among women colonized with GBS were non-African–American race (AOR = 2.13; 95% CI = 1.20–3.78) and younger age (AOR = 0.94; 95% CI = 0.91–0.98). Among GBS-colonized women with an STI in the current pregnancy, the only independent risk factor for iCLI-R was *Chlamydia trachomatis* infection (AOR = 4.31; 95% CI = 1.78–10.41).

**Conclusions:**

This study identified novel associations for GBS colonization and colonization with GBS-iCLI-R. Prospective studies will improve our understanding of the epidemiology of GBS colonization during pregnancy and the role of antibiotic exposure in alterations of the maternal microbiome.

## Introduction

*Streptococcus agalactiae* (Group B *Streptococcus* (GBS)) is a dynamic colonizer of the gastrointestinal and genitourinary tracts, frequently causing urinary tract infections, chorioamnionitis, postpartum endometritis, and bacteremia in pregnant women [1, 2]. GBS is also the most common cause of sepsis and meningitis in infants younger than three months of age [3]. With the near-elimination of *Streptococcus pneumoniae* and *Haemophilus influenzae* meningitis due to vaccination, GBS is now the most frequent cause of meningitis in childhood [4, 5]. Approximately 50% of infants born to GBS-colonized mothers acquire GBS in utero or during parturition, with 1–2% of colonized infants developing GBS disease [3]. The implementation of universal screening for GBS in pregnancy and use of intrapartum antibiotic prophylaxis (IAP) reduced the incidence of early-onset GBS disease in the United States from 1.8 cases per 1000 live births to 0.26 cases per 1000 live births from the early 1990’s to 2010 [6]. However, the incidence of late-onset GBS has remained unaffected, with ~ 0.26 cases per 1000 infants diagnosed annually [6]. As such, understanding the risk factors for maternal GBS colonization continues to be an important facet of the development of new strategies to improve maternal and neonatal outcomes.

We previously reported high rates of GBS colonization and high rates of inducible clindamycin resistance (iCLI-R) among GBS isolates from women who received obstetric care at LSUHSC-Shreveport [7]. Because of the high rates of sexually-transmitted infections (STIs) in our population and the common resistance mechanisms for iCLI-R that have been described for GBS and *Staphylococcus aureus*, we hypothesized that exposure to antibiotics during pregnancy may be a risk factor for colonization with GBS and/or colonization with a GBS strain that displays an iCLI-R phenotype. To address this question we analyzed the clinical and demographic data from the medical records of the group of women on which our earlier report was based.

## Methods

The LSUHSC-S Institutional Review Board for Human Subjects Research approved this study protocol prior to data collection.

### Study design

We identified pregnant women who were screened at 35–37 weeks gestation by vagino-rectal swab for GBS, at prenatal visits between 1 July 2009 and 31 December 2010 [7]. Antimicrobial susceptibility testing, including tests for the presence of inducible resistance to clindamycin, was performed on all GBS isolates from vagino-rectal swabs received by the UH-S Microbiology laboratory personnel according to Clinical Laboratory Standards Institute (CLSI) guidelines. We analyzed a total of 1522 medical records for pertinent clinical and demographic data. Subjects were excluded from the analyses if their records were not obtainable after more than four months of requests (n = 8) or if they were employees or spouses of employees involved in the study (n = 1) resulting in data on 1513 pregnant women available for analysis in the study population. The main outcomes of interest were: (1) GBS positivity and (2) iCLI-R among GBS positive women. We studied the relationship between each of our outcomes and antibiotic use in pregnancy (exclusive of intrapartum antimicrobial prophylaxis for GBS colonization), considering both specific antibiotics and antibiotic class. We defined “sexually-transmitted infections (STI) during the current pregnancy” as *C. trachomatis* (CT), *Neisseria gonorrheae* (NG) or *Trichomonas vaginalis* (TV), as confirmed by routine clinical testing at LSUHSC-S.

### Statistical analyses

The chi-square test was used to identify categorical variables (e.g. antibiotic exposure by class and individually, STI during pregnancy (CT, NG and TV individually and as a composite variable) significantly associated with GBS colonization and those significantly associated with iCLI-R among GBS positive women. Each antibiotic and antibiotic class was considered separately for association with GBS colonization. The two-sample t-test was used to determine if continuous variables (e.g., age, body-mass index (BMI), gravidity) are significantly associated with GBS colonization for all subjects and with iCLI-R among GBS positive women, as well as among GBS positive women with STI. Multiple logistic regression analysis was used to determine independent risk factors for GBS colonization as well as risk factors for iCLI-R among GBS positive women and among GBS positive women with STI. Our strategy for the multiple logistic regression analysis was to use as independent predictors those found to be significantly associated with the outcome from univariate analysis. We likewise used it for our post hoc analysis to determine risk factors for GBS among women with STI.

Missing data were excluded from the statistical analyses, as the percentage of missing data for the different observed variables accounted for only 0.1–6.2% of the records available for analysis, except for “GBS in prior pregnancy,” where 46.3% were appropriately “not applicable” for primigravid mothers. Data on the number of missing information for each variable are reported in the tables.

## Results

### Demographic and clinical characteristics of the study population

The characteristics of subjects (n = 1513) whose records were included in these analyses are detailed in Table 1. A majority of subjects were African–American (AA) (74.7%) and had a term delivery (91.4%). Nearly 35% had GBS colonization, 37% had a history of STI, and 55.1% received antibiotic treatment during pregnancy. The median age at the time of delivery was 23.0 years and that of the median BMI was 31.3 kg/m^2^. We tested the demographic and clinical factors listed in Table 1 for association with GBS colonization in the current pregnancy.

**Table 1:** Characteristics of Pregnant Women in Study (N = 1513)

Characteristic	Number (%)^a^ Or mean ± SD, median, range^a^	Number Unknown/NA (%)
GBS colonization	526 (34.8)	2 (0.1)^b^
STI during this pregnancy	536 (37.0)	64 (4.2)
* C. trachomatis*	332 (22.9)	64 (4.2)
* N. gonorrhea*	117 (8.1)	64 (4.2)
* T. vaginalis*	296 (20.4)	64 (4.2)
Antibiotic exposure^c^	786 (55.1)	87 (5.8)
HIV positive	14 (0.1)	54 (3.6)
Term delivery	1343 (91.4)	44 (2.9)
GBS in prior pregnancy	189 (23.2)	700 (46.3)—NA
Race		22 (1.5)
African–American	1114 (74.7)	
White	201 (13.5)	
Hispanic	141 (9.5)	
Other	35 (2.3)	
Age (years)	24.4 ± 5.8, 23.0, 13–49	18 (1.2)
Weight (lbs)	191.6 ± 49.8, 182.0, 96–408	69 (4.6)
Height (in)	64.0 ± 2.9, 64.0, 49–74	94 (6.2)
BMI	32.8 ± 7.9, 31.3, 18.8–70.1	94 (6.2)
Gravidity	2.7 ± 1.8, 2.0, 1–20	2 (0.1)

^a^Calculated from non-missing values

^b^Among the 1513 pregnant women, 2 have missing values for GBS and these 2 women have STI. The STI of these 2 women cannot be correlated to their unknown GBS. For purposes correlating STI and GBS, they are among the 66 with missing values (Table 2)

^c^Includes all antibiotic treatments for any infections diagnosed during the pregnancy, excluding IAP

### Risk factors for GBS colonization in pregnancy

African–Americans, those with STI, antibiotic exposure, and who were GBS positive in a prior pregnancy had significantly higher GBS colonization rates in the current pregnancy than those with no STI, no antibiotic exposure, non-AA race and no GBS in a prior pregnancy (Table 2a). Among continuous variables, only BMI had significant association with GBS colonization. GBS positive women had higher BMI than GBS negative women (33.7 ± 8.2 vs. 32.3 ± 7.8, *p* < 0.01). Age and gravidity were similar between GBS-positive and GBS-negative women. Although BMI was significantly associated with GBS in univariate analysis, we did not include it as a predictor variable in the multiple logistic regression model because of its significant association with AA race and because of the imprecision of BMI calculations that are not based on pre-pregnancy weights. AA women had higher BMI than non-AA women, but pre-pregnancy weights within one year prior to initiation of prenatal care were only available for a minority of subjects (data not shown). Independent risk factors for GBS colonization were STI during pregnancy and AA race as determined by multivariate analysis (Table 2b). The odds for GBS colonization among AA women was 2.142 times the odds of GBS colonization for non-AA women, while the odds of GBS colonization for women with STI were 1.309 times the odds for women without STI during pregnancy. Using the Pearson test for goodness-of-fit, the combination of AA race and STI yielded the highest *p*-value, suggesting this combination of factors provides the most accurate model of risk factors for GBS colonization. The characteristics of the 526 women with GBS colonization are summarized in Table 3.

**Table 2:** Factors associated with GBS colonization (N = 1513). (a) Categorical factors and continuous variables associated with GBS by univariate analysis—number (%) or mean ± SD, range. (B) Independent risk factors for GBS by multivariate analysis—adjusted odds ratios

Characteristic	GBS positive	GBS negative	*p* value	# Missing/NA
(a)				
*Categorical variables*				
STI during pregnancy	219/494 (44.3)	315/953 (33.1)	< 0.01**	66
Antibiotic exposure	313/488 (64.1)	471/936 (50.3)	< 0.01**	89
AA race	519/519 (86.7)	662/970 (68.2)	< 0.01**	24
GBS in prior pregnancy	80/268 (29.9)	109/544 (20.0)	< 0.01**	701
*Continuous variables*				
Age	24.1 ± 5.6, 14–44 (n = 520)	24.6 ± 5.9, 13–49 (n = 973)	0.13	10
BMI	33.7 ± 8.2, 19.4–70.1 (n = 491)	32.3 ± 7.8, 19–69 (n = 928)	< 0.01**	94
Gravidity	2.7 ± 1.8, 1–12 (n = 519)	2.7 ± 1.9, 1–20 (n = 978)	0.97	16

Characteristic	Adjusted OR	95% CI for AOR	*p* value	# Used
(b)				
African–American race	2.142	2.092–3.861	< 0.01**	
STI during pregnancy	1.309	1.035–1.653	0.02*	
Goodness of fit (Pearson test)			0.45	1432

*CI* confidence interval

^*^Significant association with GBS at 5% level of significance (*p* value < 0.05)

^**^Significant association with GBS at 1% level of significance (*p* value < 0.01)

**Table 3:** Characteristics of GBS colonized women (N = 526)

Characteristic	Number (%)^a^ Or mean ± SD, median, range^a^	Number Unknown/NA (%)
iCLI-R	124 (23.6)	–
STI during pregnancy	220 (44.5)	32 (6.1)
Antibiotic exposure	313 (64.1)	38 (7.2)
HIV positive	5 (1.0)	24 (4.6)
Term delivery	461 (90.0)	14 (2.7)
GBS in prior pregnancy	80 (29.8)	258 (49.0)—NA
AA race	450 (86.7)	7 (1.3)
IAP	384 (80.2)	47 (8.9)
Age (years)	24.1 ± 5.6, 23.0, 14–44	6 (1.1)
BMI	33.7 ± 8.2, 32.4, 19.4–70.1	35 (6.7)
Gravidity	2.7 ± 1.8, 2.0, 1–12	7 (1.3)

^a^Calculated on non-missing values

### Risk factors for iCLI-R among women with GBS strains

Only race and age were significantly associated with iCLR among the GBS colonized women in univariate analyses (Table 4a). Non-AA women had a higher rate of colonization with GBS-iCLR than AA women (33.3% vs. 22.2%, *p* = 0.04). Women with GBS-iCLR were younger than those without GBS-iCLR (23.0 ± 5.1 vs. 24.4 ± 5.8, *p* = 0.01). Race and age were also the independent risk factors for iCLR among GBS positive women as determined by multivariate analysis (Table 4b). Adjusted for age, the odds for colonization with GBS-iCLR for non-AA women were 2.13 times the odds for AA women. For every year increase in age, the adjusted odds for GBS-iCLR decreased by 0.06.

**Table 4:** Factors associated with GBS-iCL-R among GBS+ women (N = 526). (a) Categorical factors and continuous variables associated with GBS-iCL-R by univariate analysis—number (%) or mean ± SD, range. (b) Independent risk factors for iCL-R among GBS+ women by multivariate analysis

Characteristic	GBS-iCL-R Pos	GBS-iCL-R Neg	*p* value	Missing/NA
(a)				
*Categorical variables*				
STI during pregnancy	52/113 (46.0)	168/381 (44.1)	0.72	32
Antibiotic exposure	68/111 (61.3)	245/377 (65.0	0.47	38
AA race	100/123 (81.3)	350/396 (88.4)	0.04*	7
IAP	86/109 (78.9)	298/370 (80.5)	0.71	47
*Continuous variables*				
Age (years)	23.0 ± 5.1, 15–42 (n = 124)	24.4 ± 5.8, 14–44 (n = 396)	0.014*	4
BMI	34 ± 8.2, 19.4–66.1 (n = 115)	33.6 ± 8.3, 20–70.1 (n = 376)	0.67	35
Gravidity	2.6 ± 1.8, 1–9 (n = 122)	2.7 ± 1.8, 1–12 (n = 397)	0.33	7

Factor	Adjusted OR	95% CI for AOR	*p* value	# Used
(b)				
Non-AA race	2.131	1.201–3.782	< 0.01**	
Age	0.943	0.905–0.982	< 0.01**	
Goodness of fit (Pearson)			0.60	515

^*^Significant Association with iCL-R at 5% level (0.01 < *p* value < 0.05)

^**^ Significant Association with iCL-R at 5% level (*p* value < 0.01)

Forty-two percent of the GBS-iCLR cases (52 of 124) were seen in women with STI, so risk factors for iCLR were analyzed post hoc among the subgroup of GBS-positive women who had STI in the current pregnancy (Table 5). Among the 220 women with STI, 62.3% were infected with *C. trachomatis* (CT) and 99.5% were treated with antibiotics. Among the 213 women treated with antibiotics, more than half (132 out of 213, 62%) received azithromycin (AZ). Categorical factors significantly associated with GBS-iCLR colonization were CT infection and AZ treatment (Table 6a). GBS-iCLR rates were significantly higher in women with CT and those treated with AZ than those without CT and those treated with antibiotics other than AZ (32.8% and 32.6% vs. 8.4% and 8.6%, respectively; *p* < 0.01). Among continuous variables, only age was significantly associated with GBS-iCLR colonization (Table 6a). Women with GBS-iCLR were significantly younger than those without GBS-iCLR (21.2 ± 3.7 years vs. 23.4 ± 5.2 years, *p* < 0.01). Thus, factors significantly associated with iCLI-R by univariate analysis were CT infection, AZ treatment and younger age. The only independent risk factor for colonization with GBS-iCLR among GBS-positive women with STI was CT infection (Table 6b). Adjusted for age and AZ treatment (factors associated with GBS-iCLR by univariate analysis), the odds for iCLR among women with CT were 4.31 (95% CI = 1.78–10.41) times the odds among those without CT. Although age and AZ treatment had significant associations with iCLR by univariate analysis, they dropped out as risk factors for iCLR in multivariate analysis because of their significant associations with CT infection (*p* < 0.01).

**Table 5:** Characteristics of GBS Positive Women with STI (N = 220)

Characteristic	Number (%)^a^ Or mean ± SD, median, range^a^	Number Missing/NA (%)
iCLI-R	52 (23.6)	–
*Chlamydia trachomatis* (CT)	137 (62.3)	–
Antibiotic exposure	213 (99.5)	6 (2.7)
AZ treatment	132 (62.0)	7 (3.2)
AA race	208 (94.6)	–
Term delivery	198 (92.5)	6 (2.7)
IAP	171 (82.2)	12 (5.5)
HIV positive	0 (0)	10 (4.4)
GBS in prior pregnancy	36 (29.3)	97 (44.0)
Age (years)	22.9 ± 5.0, 22.0, 15–41	3 (1.4)
BMI	32.9 ± 8.0, 31.6, 19.4–62.3	12 (5.5)
Gravidity	2.6 ± 1.8, 2.0, 1–10	1 (0.5)

^a^Calculated from non-missing values

**Table 6:** Factors associated with GBS-iCL-R among GBS positive women with STI (N = 220). (a) Factors associated with GBS-iCL-R among GBS positive women with STI by univariate analysis—number (%) or mean ± SD, range. (b) Independent risk factor for iCL-R among GBS+ women with STI by multivariate analysis—adjusted odds ratio

Characteristic	GBS-iCL-R Pos	GBS-iCL-R Neg	*p* value	Missing/NA
(a)				
*Categorical variables*				
CT infection	45/52 (86.5)	92/168 (54.8)	< 0.01**	–
AZ treatment	43/50 (86.0)	89/163 (54.5)	< 0.01**	7
*Continuous variables*				
Age	21.2 ± 3.7, 15–34 (n = 52)	23.4 ± 5.2, 15–41 (n = 165)	< 0.01**	3
BMI	31.3 ± 6.0, 19.4–45.4 (n = 48)	33.4 ± 8.5, 20–62.3 (n = 160)	0.06	12
Gravidity	2.5 ± 2.0, 1–9 (n = 52)	2.6 ± 1.7, 1–10 (n = 167)	0.85	1

Factor	Adjusted OR	95% CI for AOR	*p* value	# Used
(b)				
CT infection	4.31	1.78–10.41	< 0.01	
Goodness of fit test			0.79	217

## Discussion

In this retrospective study, we correlated clinical and microbiologic data from a large population of pregnant women in order to identify risk factors for maternal colonization with GBS and GBS-iCLR. We hypothesized that antibiotic exposure in pregnancy, particularly macrolide exposure, is associated with colonization with GBS strains with inducible resistance to clindamycin (GBS-iCLR) due to selective pressure on the maternal flora. Our analyses revealed that GBS colonization in pregnant women was significantly associated with STI in the current pregnancy, a novel finding, as well as AA race, a previously known risk factor. The association of GBS-iCLR with non-African–American race and younger age and the association of GBS-iCLR with CT infection and azithromycin exposure among women with an STI in the current pregnancy are unique findings that suggest numerous hypotheses to be tested in future, prospective studies.

Prior studies have identified numerous risk factors for GBS infection in infants, including pre-term delivery, prolonged rupture of membranes, maternal chorioamnionitis, maternal colonization with GBS during labor and delivery, multiparity, maternal GBS colonization in a previous pregnancy, low maternal levels of anti-GBS antibody, certain maternal sexual behaviors, African–American race and GBS bacteriuria during pregnancy, many of which are also risk factors for maternal colonization during a subsequent pregnancy [8–14]. However, relatively few studies have explicitly focused on risk factors for maternal colonization with GBS or correlated microbiologic and clinical data for large cohorts of patients. The landmark VIP study from 1984 to 1989 did not find an association of GBS colonization with STIs, but the rates of GBS colonization and STIs were significantly lower in that multi-center study and variable between the geographically distributed study sites [15]. Colonization with GBS in a prior pregnancy has been identified as a risk factor for GBS colonization in a subsequent pregnancy by several studies [9–11, 16]. African–American race, age > 21 years and marijuana use within the past four months were identified as risk factors for rectal GBS colonization among non-pregnant women in a longitudinal study [17]. The same study found that recent vaginal intercourse, vaginal yeast or *E. coli* colonization and abnormal vaginal flora (Nugent score ≥ 4), but not STI, hormonal contraception or specific sexual practices, were associated with vaginal GBS colonization among young adult women [17]. The study by Rocchetti and colleagues identified frequency of sexual intercourse, alterations in vaginal flora and prior miscarriage as risk factors for GBS colonization in pregnancy [18].

Studies comparing the vaginal flora of GBS+ and GBS− women in pregnancy have yielded conflicting results, leaving uncertainty about the importance of alterations of the vaginal microbiome as a contributing or protective factor in GBS colonization [19, 20]. Recent studies failed to detect an association between GBS and lactobacilli in pregnant Polish and Guatemalan women using culture-based and molecular testing, respectively [20, 21]. A large study of the vaginal microbiome in non-pregnant women found strong positive and negative associations between GBS colonization and several bacterial taxa, but no association between GBS and *Lactobacillus* species was found, suggesting that vaginal microbiome community state type is not a reliable predictor of GBS colonization in non-pregnant women [22]. Longitudinal studies of pregnant women have begun to characterize the diversity and dynamics of the vaginal microbiome and identified unique community state types and, more recently, the relative abundance of various *Lactobacilli* as important correlates of premature delivery [23, 24]. These emerging studies and novel research modalities suggest the potential use of non-culture techniques to assess risk for adverse maternal and infant outcomes.

While all known GBS isolates retain susceptibility to penicillin in vitro, GBS isolates with resistance to erythromycin and clindamycin have been increasingly reported worldwide, including at our institution [7, 25–27]. Furthermore, GBS isolates with reduced penicillin susceptibility have been identified at several sites around the world, highlighting the need for continued active surveillance for alterations in GBS susceptibility that may again alter the strategies for prevention of GBS disease [28, 29]. The identification of clinical factors associated with changing antimicrobial susceptibility profiles, especially antibiotic exposures, is critical to understanding the role of antimicrobial pharmacodynamics in the promotion of antibiotic resistance in commensal and opportunistic microbes that impact maternal and infant outcomes, especially for drugs like azithromycin that have long half-lives and are used in large doses for treatment of *C. trachomatis* infection [30, 31]. Furthermore, the consideration of using wide-spread prophylactic dosing of azithromycin for prevention of adverse maternal, neonatal and infant outcomes in underdeveloped countries portends a significant risk of promoting antibiotic resistance in areas of the world that are least-equipped to combat multi-drug resistant organisms [32, 33].

This study has several notable strengths, including the correlation of microbial and clinical data from a large study population, and the identification of novel clinical variables associated with GBS and GBS-iCLR colonization. The predominance of African–Americans in the study is also a strength, given that few studies have focused on this population previously. Weaknesses of this study include the lack of data on GBS serotypes, multi-locus sequence types, and molecular characterization of resistance genes prevalent in the GBS isolates, all of which were not possible in the present study, but should be performed in future prospective studies. Additionally, the lack of information regarding maternal sexual, dietary, social and hygiene practices limits the comparability of these results to some prior studies and precludes the evaluation of some potentially confounding factors.

The association of GBS-iCLI-R with *C. trachomatis* infection and azithromycin exposure supports the hypothesis that azithromycin exerts selective pressure on GBS, potentially through the *erm* genes that mediate Macrolide–Lincosamide–Streptogramin resistance (MLS_**B**_ resistance) in GBS and staphylococci [34, 35]. Erythromycin and clindamycin-resistant Gram-positive organisms often carry erythromycin ribosomal methylation (*erm*) genes accounting for constitutive (cMLS_B_-phenotype) and inducible (iMSL_B_-phenotype) resistance in GBS isolates [36]. Genes contributing to erythromycin resistance include *ermA(TR)*, *ermB*, and *mefA*, which have been found among macrolide-resistant GBS isolates [25, 37–39]. In a large collection of GBS isolates from numerous sites in Brazil, erythromycin resistance was not associated with particular GBS serotypes, suggesting the independent acquisition of erythromycin resistance genes [37]. Prospective molecular analysis will be necessary to determine the precise genetic mechanism(s) that mediate iCLI-R in our population and the potential changes over time in resistance patterns. These data support a particular focus on antimicrobial stewardship and infection control practices in pregnancy and the need for prospective studies to characterize the impact of antibiotic exposures on the maternal microbiome. Furthermore, they support the need for intensive education of patients and providers regarding prevention of STIs in pregnancy, especially in communities with high rates of STIs.

## Conclusion

In summary, this large retrospective study of clinical and microbiological data identified STI potential risk factor for GBS colonization in pregnancy in a population with high rates of STIs. Additionally, the receipt of azithromycin during pregnancy was significantly associated with colonization due to a GBS strain with inducible clindamycin resistance among women with STI in the current pregnancy. These findings have significant implications for the promotion of antimicrobial resistance, especially when public health interventions include widespread use of antibiotics. Prospective studies will be necessary to correlate molecular mechanisms of antibiotic resistance, GBS serotypes and genotypes, clinical factors and changes in the maternal microbiome with maternal and neonatal outcomes and to determine the likelihood that antimicrobial resistance will persist in communities.

## Data Availability

The datasets generated during and/or analyzed during the current study are not publicly available due to inclusion of Protected Health Information but are available from the corresponding author in de-identified form on reasonable request.
